# Association between polymorphisms in *NOS3* and *KCNH2* and social memory

**DOI:** 10.3389/fnins.2015.00393

**Published:** 2015-10-21

**Authors:** Susanne Henningsson, Anna Zettergren, Daniel Hovey, Lina Jonsson, Joakim Svärd, Diana S. Cortes, Jonas Melke, Natalie C. Ebner, Petri Laukka, Håkan Fischer, Lars Westberg

**Affiliations:** ^1^Department of Pharmacology, Institute of Neuroscience and Physiology at the Sahlgrenska Academy, University of GothenburgGothenburg, Sweden; ^2^Department of Psychiatry and Neurochemistry, Institute of Neuroscience and Physiology at the Sahlgrenska Academy, University of GothenburgGothenburg, Sweden; ^3^Aging Research Center, Karolinska InstituteStockholm, Sweden; ^4^Department of Psychology, Stockholm UniversityStockholm, Sweden; ^5^Department of Psychology, University of FloridaGainesville, FL, USA; ^6^Department of Aging and Geriatric Research, University of FloridaGainesville, FL, USA

**Keywords:** face recognition, social, memory, nitric oxide, NOS3, KCNH2

## Abstract

Social memory, including the ability to recognize faces and voices, is essential for social relationships. It has a large heritable component, but the knowledge about the contributing genes is sparse. The genetic variation underlying inter-individual differences in social memory was investigated in an exploratory sample (*n* = 55), genotyped with a chip comprising approximately 200,000 single nucleotide polymorphisms (SNPs), and in a validation sample (*n* = 582), where 30 SNPs were targeted. In the exploratory study face identity recognition was measured. The validation study also measured vocal sound recognition, as well as recognition of faces and vocal sounds combined (multimodal condition). In the exploratory study, the 30 SNPs that were associated with face recognition at *p*_uncorrected_ < 0.001 and located in genes, were chosen for further study. In the validation study two of these SNPs showed significant associations with recognition of faces, vocal sounds, and multimodal stimuli: rs1800779 in the gene encoding nitric oxide synthase 3 (*NOS3*) and rs3807370 in the gene encoding the voltage-gated channel, subfamily H, member 2 (*KCNH2*), in strong linkage disequilibrium with each other. The uncommon alleles were associated with superior performance, and the effects were present for men only (*p* < 0.0002). The exploratory study also showed a weaker but significant association with (non-emotional) word recognition, an effect that was independent of the effect on face recognition. This study demonstrates evidence for an association between *NOS3* and *KCNH2* SNPs and social memory.

## Introduction

Social memory refers to the ability to recognize the identity of previously encountered individuals, an ability essential for successful social interactions. While in rodents this skill is based on olfactory and pheromonal signals, in humans it is based mainly on the identification of faces and voices (Belin et al., 2011).

Face recognition ability, the most investigated facet of social memory in humans, varies considerably in the population (Kennerknecht et al., 2006; Russell et al., 2009) and has been reported to be highly heritable (Wilmer et al., 2010), indicating that some of the inter-individual variation in face recognition ability can be explained by genetic factors. For prosopagnosia—characterized by serious impairments in face recognition (Hecaen and Angelergues, 1962; Benton and Van Allen, 1972)—an autosomal dominant inheritance has even been suggested (Kennerknecht et al., 2006).

To date, the knowledge about which molecules are involved in social memory is sparse. Sex differences with regard to the ability to recognize faces and the mechanism for processing faces (Fischer et al., 2007) have provided some evidence for involvement of molecules related to sex differentiation. So far, rodent studies have put forward oxytocin (Ferguson et al., 2000, 2001), vasopressin (Le Moal et al., 1987), estrogen (Choleris et al., 2003), and nitric oxide (Mutlu et al., 2011) as crucial players for social memory, suggesting related genes to be candidates in humans (Skuse et al., 2014).

Domain-specificity has been suggested for recognition of faces, as compared to objects (Rezlescu et al., 2014; Weigelt et al., 2014). In line with this, face memory deficits have been shown often to be associated with a tendency to process faces as if they were any other object (Langdell, 1978; Boucher and Lewis, 1992; Rutherford et al., 2007; Spezio et al., 2007; Adolphs et al., 2008; Harms et al., 2010; McPartland et al., 2011; Arkush et al., 2013).

With the aim to identify common genetic variation that influences the mechanism of, and explain the differences in, social memory, we investigated two independent samples of healthy men and women. A first exploratory study (*n* = 55) measured performance in recognition of neutrally expressive faces. In this first study, we also had access to performance on a task measuring word memory, thus enabling analyses of whether associations with memory deficits were face-specific or rather due to an effect on general memory. A second, validation study (*n* = 582) measured performance in recognition of faces displaying both neutral and emotional expressions, as well as recognition of neutral and emotional vocal sounds and recognition in a multimodal condition where participants saw faces and heard the corresponding sounds simultaneously. Of the approximately 200,000 single nucleotide polymorphisms (SNPs) genotyped with a chip (the MetaboChip) in the exploratory study, 30 were filtered out as associated (*p* < 0.001) with the ability to recognize faces, and were subsequently targeted in the validation study.

## Materials and methods

### Participants

#### Exploratory study

Social memory performance and genetic data were available for a total of 55 participants, 29 women and 26 men (25 younger: 20–31 years, mean ± sd: 25.1 ± 3.4; 12 females; 30 older: 65–74 years, mean ± sd: 68.2 ± 2.5; 17 females; Ebner et al., 2012). Word memory data was available for 56 participants (29 women, 27 men). All participants were right-handed, native Swedish speakers, had normal or corrected-to-normal vision, had no contraindications to magnetic resonance imaging (MRI), had no history of stroke, heart disease or primary degenerative neurological disorder, no past or present neuropsychiatric diseases, diabetes or neurological disorders, and were free from blood-thinning medication, as assessed by self-reported medical history. For older adults, a radiologist screened a T1-weighted and a T2-weighted image and ruled out abnormal levels of atrophy or lesions. All participants provided written informed consent in accordance with the Declaration of Helsinki. The study was approved by the regional ethical review board of Stockholm. All participants were Caucasian, as indicated by self-report.

#### Validation study

The validation study included 582 participants for whom both behavioral and genetic data were available, 223 men (age range: 18–36 years, mean ± sd: 23.4 ± 3.3) and 359 women (age range: 18–34 years, mean ± sd: 22.9 ± 3.2). All participants were right-handed, fluent in Swedish, healthy and had no past or present psychiatric diseases, or substance abuse. All participants provided written informed consent in accordance with the Declaration of Helsinki. The study was approved by the regional ethical review board of Stockholm. Ethnicity was assessed by asking which country parents and grand-parents were born in. Eighty-seven percent (181 men, 309 women) of the participants were Caucasian.

### Genotyping

#### Exploratory study

The participants were genotyped using the Illumina iSelect MetaboChip. This chip includes nearly 200,000 SNPs selected from the results of genome-wide meta-analyses of several metabolism- and cardiovascular-relevant traits. It was designed to capture genetic variation coupled to type two diabetes, coronary artery disease, and myocardial infarction (Voight et al., 2012). Cardiovascular-relevant genes are often important for general brain function. The MetaboChip thus also covers genomic regions of interest for memory and face processing, including those involved in hormonal and neurotransmitter functions. Moreover, as the chip covers most of the genome, further polymorphisms relevant for social memory are covered in an indirect way by being in high linkage disequilibrium (LD) with those on the chip.

#### Validation study

DNA was extracted from saliva samples using OraGene DNA self-collection kit (DNA Genotek, Inc., Ottawa, ON, Canada). The 30 SNPs that showed an association with face recognition at *p* < 0.001 in the exploratory study were genotyped with KASPar®, a competitive allele-specific polymerase chain reaction SNP genotyping system using FRET quencher cassette oligos (http://www.lgcgenomics.com). The genotyping success rate was >95% and all of the SNPs were found to be in Hardy Weinberg equilibrium. The association observed for the *NOS3* polymorphism (see Results) prompted us to genotype two additional SNPs in this gene, rs2070744 and rs1799983.

### Tasks in the exploratory study

#### Face recognition task

Face recognition was measured during functional MRI (fMRI) scanning (see Ebner et al., 2012 for an fMRI study on this sample). During incidental encoding (8.4 min), 48 photographs of neutral faces (unique face identities) were presented in pseudo-random order, and interspersed with 24 low-level null events (black crosses on gray background). Each stimulus was shown for 3.5 s. In between two faces, a fixation cross appeared on the screen in a jittered fashion (3–4 s). The pictures were taken from the FACES database (Ebner et al., 2010) and depicted equal numbers of men and women and younger and older faces. The instruction was to look at the pictures “as if you watched TV.” After a ten-min retention interval, during which anatomical images were taken, the two fMRI runs (each 8.4 min) of a surprise recognition task followed, during which the same 48 faces were presented at the same rate as at encoding (but in a different, pseudo-randomized order), together with 48 new distractor faces, randomly interspersed. The task was to indicate whether a face had been previously seen or not via button press (Gardiner and Richardson-Klavehn, 2000). D prime (d') was calculated based on participants' responses (MacMillan and Creelman, 2005).

#### Word recognition task

Word pair recognition memory was administered using the category-instance task (Dolan and Fletcher, 1997; Nyberg et al., 2009). Prior to scanning, participants were presented with pairs of words (*n* = 34) consisting of a category (e.g., “tree”) and an instance of the category (e.g., “pine”). Participants were instructed to memorize the pairs. Each pair was presented twice and for 2 s. During fMRI scanning, another list of word pairs was presented (2 s/item) with a jittered (1–6 s) inter-stimulus display of a fixation cross. A third of the pairs of this list had been presented before (old category, old instance; *n* = 17), another third were new (new category, new instance; *n* = 17), and the last third were categories that had been seen before but were paired with new instances (old category, new instance; *n* = 17). Participants again were instructed to memorize the pairs. After a 50 min retention interval, during which participants performed other tasks in the scanner, a cued recall test was administered outside the scanner. During this recall test, participants were presented with all categories seen during the task and were asked to pair each category with the instance presented during scanning. Based on these responses, the sum of correct responses was computed as a measure of word pair recognition memory.

### Task in the validation study

#### Social memory task

The social memory task, measuring the recognition of the identity of faces, vocal sounds and their combination, consisted of an incidental encoding session and a recognition session. In contrast to the exploratory study, the validation tasks were not performed in a scanner but measured behaviorally only. During the encoding session, participants were presented with 24 photographs of faces, followed by 24 human vocal sounds, followed by 24 multimodal stimuli (photographs of faces presented together with vocal sounds). The order was randomized (within the modality) across subjects and no face or voice identity was presented more than once in each condition. In contrast to the first exploratory study, the faces and voices expressed anger, disgust, fear, happiness, sadness, or no emotion (four stimuli for each condition). The timing for the presentation was self-paced and participants were asked to indicate with the mouse which emotion was conveyed by the stimulus in a forced-choice task. The response options were the same as the expressed emotions. MediaLab software (Jarvis, 2008) was used for stimulus presentation and recording of responses. The pictures were color photographs from the FACES database (Ebner et al., 2010) and the sounds were non-linguistic emotional vocalizations (e.g., crying, laughter, sighs, screams) from the VENEC database (Laukka et al., 2013). At recognition (6–10 min after encoding depending on condition and reaction times), the same stimuli, interspersed with the same number of new distractor stimuli (24 old, 24 new), were presented. The same identity always expressed the same emotion. Participants were asked to indicate (self-paced) whether they recognized a stimuli or not, using the remember/know-paradigm (Gardiner and Richardson-Klavehn, 2000). Pooling the correct answers (remember and know answers) and controlling hit frequency for false alarms (Stanislaw and Todorov, 1999) provided four measures of recognition accuracy: d'faces, d'vocal, d'multimodal, and the average across presentation modalities, d'all.

### Procedure

In the exploratory study, associations between nearly 200,000 SNPs and face recognition were explored. The most promising variants were further analyzed in the validation study with regard to a potential association with the d'all measure of social memory. *Post-hoc* analyses for significant SNPs included the specific measures d'faces, d'vocal, and d'multimodal. To determine whether the association with memory was specific for the social dimension, *post-hoc* analyses also included the word pair recognition task performed by the participants in the exploratory study.

### Statistical analyses

Linear regressions using SNP and Variation Suite v7.7 (Golden Helix, Inc., Bozeman, MT, www.goldenhelix.com) were used to determine the significance levels of the MetaboChip SNPs with MAF>5%. SPSS (IBM Corp., Version 22.0. Armonk, NY) was used for further examination of the *NOS3* and *KCNH2* associations in the exploratory study and for the 30 SNPs in the validation study. Linear regression models (additive model) were used, treating the heterozygote as the intermediate. Multiple testing was controlled for using Bonferroni correction. The significance level for the validation study was thus set to *p* = 0.00083 (0.05/(30 SNPs ^*^ 2 sexes)). Linkage disequilibrium (LD) between polymorphisms was assessed by Haploview 4.2 (Barrett et al., 2005).

## Results

### Exploration and validation of associations between SNPs and social memory

None of the 200,000 SNPs genotyped in the exploratory study displayed an association with face recognition memory that survived correction for multiple testing. Thirteen SNPs were significant at a threshold of *p* < 0.0001, 113 at a threshold of *p* < 0.001 and 1507 at a threshold of *p* < 0.01. Of the 113 SNPs showing suggestive evidence of association at a level of *p* < 0.001, those that had a minor allele frequency (MAF) > 5%, were in Hardy Weinberg Equilibrium, were located in genes, and were not in high LD (*r*^2^ > 0.8) with SNPs in the same gene, were filtered out. Table 1 shows these 30 SNPs, which were also analyzed in the validation study with regard to their association with the d'all measure. Two of the SNPs, the rs1800779 in *NOS3* and the rs3807370 in *KCNH2*, in high LD (*D*' > 0.9, *r*^2^ > 0.9) with each other, displayed associations that survived correction for multiple testing (see information below). None of the other 28 SNPs showed associations that survived correction for multiple testing.

**Table 1:** **Promising SNPs (*p* < 0.001) identified in the exploratory study and selected for further analyses in the validation study**.

**Gene**	**Polymorphism**	**MAF exploratory**	**MAF validation**	**Corr *p*-value validation M/F**
*B7H6*	rs61880293	0.07	0.09	ns/ns
*BEAN1*	rs893198	0.27	0.30	ns/ns
*BRE*	rs12468596	0.29	0.23	ns/ns
*C10orf90*	rs11245011	0.19	0.13	ns/ns
*DEF8*	rs17784583	0.19	0.21	ns/ns
*DGKB*	rs11767076	0.21	0.29	ns/ns
*FBXL17*	rs11242664	0.18	0.15	ns/ns
*FGF5*	rs982804	0.48	0.50	ns/ns
*GMDS*	rs2569842	0.28	0.28	ns/ns
*IGSF11*	rs251457	0.39	0.38	ns/ns
*KCNH2*	rs3807370	0.37	0.29	0.012/ns
*LOC344595*	rs1283101	0.32	0.41	ns/ns
*LPCAT1*	rs11133792	0.31	0.23	ns/ns
*LPL*	rs264	0.14	0.15	ns/ns
*MRPL17*	rs1567135	0.37	0.46	ns/ns
*NOS3*	rs1800779	0.38	0.30	0.006/ns
*PCSK5*	rs6560494	0.42	0.45	ns/ns
*PPM1B*	rs2054005	0.20	0.23	ns/ns
*PRICKLE1*	rs1452106	0.44	0.40	ns/ns
*PTPRG*	rs9856420	0.25	0.24	ns/ns
*PVRL2*	rs1871047	0.34	0.38	ns/ns
*SEPT2*	rs12694997	0.30	0.19	ns/ns
*SLC22A16*	rs2494553	0.07	0.04	ns/ns
*SLC26A7*	rs10099092	0.08	0.08	ns/ns
*SPRED2*	rs12612780	0.30	0.33	ns/ns
*SYN2*	rs62240442	0.08	0.05	ns/ns
*TTC39B*	rs581080	0.20	0.21	ns/ns
*TTC39B*	rs643531	0.14	0.13	ns/ns
*WHSC1*	rs474235	0.14	0.18	ns/ns
*ZC3H12D*	rs512685	0.15	0.18	ns/ns

MAF, minor allele frequency; ns, non-significant; corr, corrected (uncorrected p-value ^*^ 60); M, males; F, females.

### *KCNH2* and *NOS3* SNPs in the exploratory and validation studies

**Figure 1:**
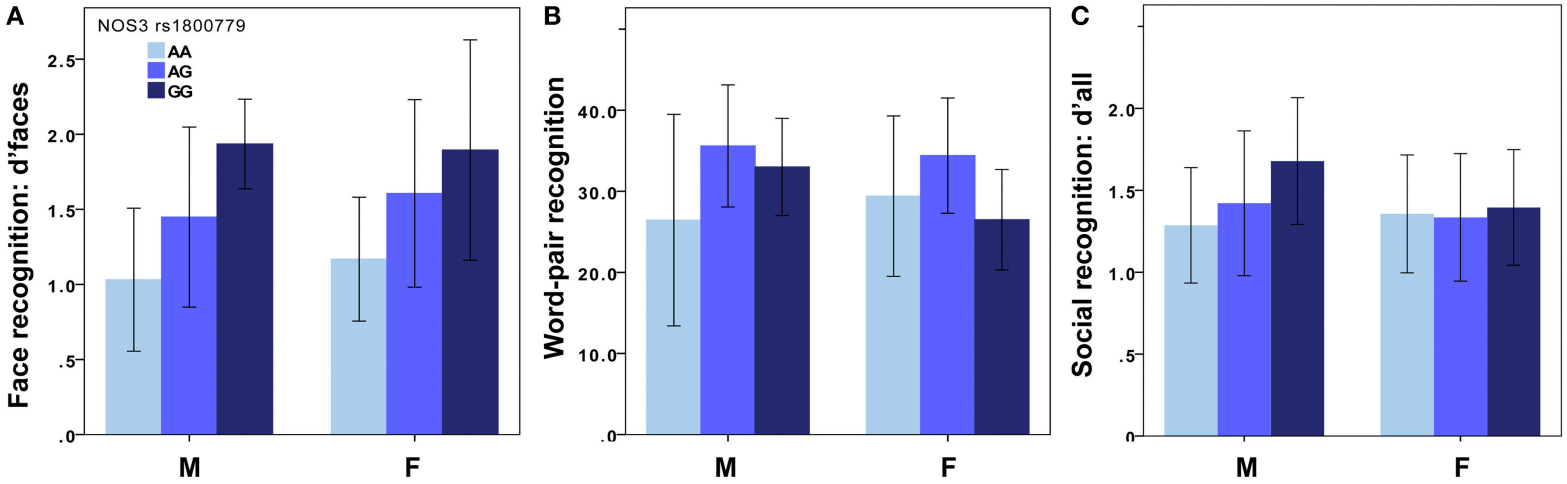
**Recognition memory for ***NOS3*** genotypes**. In the exploratory study, face **(A)** and word recognition **(B)** was superior in carriers of the G-allele of the *NOS3* rs1800779 (M: *n*_AA_ = 9, *n*_AG_ = 14, *n*_GG_ = 3 for faces and *n*_AA_ = 9, *n*_AG_ = 15, *n*_GG_ = 3 for words. F: *n*_AA_ = 11, *n*_AG_ = 15, *n*_GG_ = 3 for faces and *n*_AA_ = 10, *n*_AG_ = 15, *n*_GG_ = 4 for words). The results were similar for the *KCNH2* polymorphism. In the validation study, social recognition memory **(C)** was superior in male carriers of the G-allele of the *NOS3* rs1800779 (M: *n*_AA_ = 126, *n*_AG_ = 84, *n*_GG_ = 14. F: *n*_AA_ = 166, *n*_AG_ = 149, *n*_GG_ = 44). The results were similar for the *KCNH2* polymorphism and the *NOS3* rs2070744 polymorphism. The figure displays mean ± sd. M, Male; F, Female. See the Results Section for *p*-values.

In the exploratory study, two of the 30 SNPs showing associations with face recognition (d'faces-n) at *p* < 0.001, were rs1800779 in *NOS3* and rs3807370 in *KCNH2* (*NOS3*: *p* = 0.0006, β = 0.45; *KCNH2*: *p* = 0.0009, β = 0.43): less common homozygotes (GG and AA, respectively), showed more accurate face recognition. The effects were similar for men and women (*NOS3* men: *p* = 0.01, β = 0.49, women: *p* = 0.02, β = 0.42, Figure 1A; *KCNH2* men: *p* = 0.02, β = 0.45, women: *p* = 0.02, β = 0.42). There was no significant difference in face recognition performance between younger and older participants [*p* = 0.09, *t*_(56)_ = 1.7], nor between men and women [*p* = 0.48, *t*_(56)_ = −0.7].

In the validation study, d'all was significantly associated with both polymorphisms in men (*NOS3*: *p* = 0.0001, β = 0.25, Figure 1C; *KCNH2*: *p* = 0.0002, β = 0.25) but not in women (*NOS3*: *p* = 0.8, β = 0.014, Figure 1C; *KCNH2*: *p* = 0.75, β = 0.017). These results did not change notably when participants of non-Caucasian origin were excluded from the analyses (men: *NOS3*: *p* = 0.0001, β = 0.28; *KCNH2*: *p* = 0.00008, β = 0.29; women: *NOS3*: *p* = 0.85, β = 0.01; *KCNH2*: *p* = 0.76, β = 0.02). The associations survived correction for multiple testing (*p* < 0.00083; see Table 1 for corrected *p*-values). *Post-hoc* tests showed this effect (in males) to be present for d'faces (*NOS3*: *p* = 0.002, β = 0.20; *KCNH2*: *p* = 0.02, β = 0.16), d'vocal (*NOS3*: *p* = 0.008, β = 0.18; *KCNH2*: *p* = 0.001, β = 0.22), and d'multimodal (*NOS3*: *p* = 0.007, β = 0.18; *KCNH2*: *p* = 0.02, β = 0.16).

To further investigate the importance of *NOS3*, two additional SNPs of known functional importance were genotyped. The *NOS3* promoter SNP, rs2070744 (MAF = 0.30), showed effects very similar to the rs1800779, i.e., a significant association with d'all in men (*p* = 0.0002, β = 0.25), but not in women. As for rs1800779, *post-hoc* tests showed associations with d'faces (*p* = 0.002, β = 0.21), d'vocal (*p* = 0.008, β = 0.18) and d'multimodal (*p* = 0.01, β = 0.16) in men. Carriers of the uncommon CC genotype displayed better social recognition. The exon 8 rs1799983 (MAF = 0.27) showed a weak effect on the d'all measure in men (*p* = 0.04, β = 0.14) that did not survive correction for multiple testing. The rs2070744 showed high LD with the *KCNH2* SNP and the *NOS3* 1800779 (*D*' > 0.9, *r*^2^ > 0.9), whereas the LD between rs1799983 and the other three polymorphisms was low (*D'* < 0.5, *r*^2^ < 0.2). *T*-tests revealed that there was no significant difference in any of the measures of social memory between men and women (*p* > 0.7).

*NOS3* rs1800779 and *KCNH2* rs3807370 polymorphisms were significantly associated with word pair recognition, but only for dominant models, i.e., when pooling the uncommon genotype with the heterozygote. Carriers of the uncommon allele had better memory than carriers of the common homozygous genotype [*NOS3*: *p* = 0.02, *t*_(54)_ = 2.4, Figure 1B; *KCNH2*: *p* = 0.03, *t*_(54)_ = 2.3], an effect driven by men [*NOS3*: *p* = 0.03, *t*_(25)_ = 2.3; *KCNH2*: *p* = 0.04, *t*_(25)_ = 2.1].

The two performance measures of face (d'faces-n) and word pair recognition in the exploratory study did not correlate significantly (all: *p* = 0.08, Pearson = 0.24; men: *p* = 0.07, Pearson = 0.37; women: *p* = 0.58, Pearson = 0.11). To explore whether the genetic effects on face and word memory were dependent, word recognition performance was added as covariate to the regression models with d'faces-n as the dependent variable. The addition did not change the results, neither for the *NOS3* rs1800779 (*p* = 0.0006, β = 0.45 alone in the model; *p* = 0.0006, β = 0.45 with word memory performance in the model), nor for the KCNH2 polymorphism (*p* = 0.0009, β = 0.43 alone in the model, *p* = 0.0008, β = 0.44 with word memory performance in the model). Also, word pair recognition memory was not a significant predictor in the models.

## Discussion

Advances in the field have proven that the heritability of complex traits such as social memory is explained by a large number of common genetic variants, all contributing with very small effects. The attempt to detect new variants and genes using genome-wide approaches requires very large samples to reach the genome-wide corrected significance level. We therefore investigated the possibility to validate the most promising associations between SNPs and behavior from an exploratory study of many SNPs in a small sample—not surviving correction for multiple testing—in a larger independent sample. By using this strategy we identified the *NOS3* rs1800779 and *KCNH2* rs380730 polymorphisms, in high LD with each other, as intriguing contributors to the inter-individual variation in social memory. The associations survived correction for multiple testing in the considerably larger validation sample. Furthermore, in the exploratory sample the polymorphisms associated with face recognition independently of sex, while, in the validation study they were associated with recognition of identity through faces as well as vocal sounds in men only. The smaller exploratory study provided evidence for a weaker association also with word pair memory, an effect that was independent of the effect of social memory.

*NOS3* is situated just downstream of *KCNH2* on chromosome 7q36. The two genes, both expressed in the brain (Judas et al., 1999; Huffaker et al., 2009), encode nitric oxide synthase 3, i.e., endothelial NOS, and the potassium voltage-gated channel, subfamily H, member 2, respectively. Endothelial NOS is responsible for the production of nitric oxide (NO) from L-arginine in the endothelium. NO is vasodilatory, and regulates cerebral blood flow (Ignarro, 1989; Quyyumi et al., 1995). It triggers multiple signal transduction pathways and influences synaptic function including transmitter release (Sagi et al., 2014). NO and NOS3-mediated NO signaling is known to be involved in hippocampal long-term potentiation (LTP) (Schuman and Madison, 1991; Dinerman et al., 1994; O'Dell et al., 1994) and has therefore been investigated in relation to learning and memory. Evidence for an involvement of NOS in social memory has been provided by rodent studies showing that NOS inhibitors impair olfactory memory in a social recognition test (Böhme et al., 1993; Mutlu et al., 2011), as well as in non-social memory (Böhme et al., 1993), an effect that required inhibition of both endothelial and neuronal NOS (*NOS1*) (Mutlu et al., 2011). The current finding thus provides intriguing evidence of a conserved role for NO in modulating social cognition in humans, comparable to the conserved role shown for oxytocin (Skuse et al., 2014).

Although the uncommon allele of the *NOS3* promoter SNP rs1800779 has been associated with lower peripheric levels of NOS3 mRNA and protein (Aminuddin et al., 2013), its function is, as of yet, not established. However, the other two *NOS3* polymorphisms genotyped in the validation study, i.e., the rs2070744 and rs1799983, are known to be functional, the promoter polymorphism rs2070744 (-786T/C) affecting the expression of the gene (Nakayama et al., 2000; Kittel-Schneider et al., 2015), and the exon 8 rs1799983 (894G/T) implicating an amino acid substitution, Glu298Asp, that causes a truncation of the protein (Tesauro et al., 2000). In the present study the effect for rs2070744 was equally strong as the effect for rs1800779. Therefore, it is plausible that this SNP, in high LD with the rs1800779, is responsible for the observed association. Functional studies have consistently shown the uncommon C-allele of rs2070744, here associated with superior social recognition, to be associated with less gene expression. It has been shown to reduce promoter activity (Nakayama et al., 2000), to be associated with lower mRNA (Venturelli et al., 2005; Kittel-Schneider et al., 2015) and NO metabolite levels in blood (Kittel-Schneider et al., 2015), and with a lack of shear stress-induced *NOS3* expression (Cattaruzza et al., 2004). In line with our finding of enhanced social memory in carriers of the allele related to reduced NOS3 and thus NO production, elevated NO levels in blood have been negatively correlated with the performance on memory tests in humans (Talarowska et al., 2012).

To our knowledge, only one previous study has examined the relationship between *NOS3* and memory in humans (Solé-Padullés et al., 2004). This previous study comprised participants with mild cognitive impairment. They showed that carriers of the uncommon Asp-allele of rs1799983, resulting in a truncation of the protein, had *lower* memory performance, which is consistent with the relationship between NOS inhibition and impaired memory in rodents. In contrast, in our sample, the Asp-allele was weakly (not surviving correction for multiple testing) associated with *enhanced* social memory.

The promoter polymorphisms in *NOS3* are also in high LD with the intron 2 polymorphism in the neighboring gene *KCNH2*, which thus may be responsible for the associations reported. The rs380730 is located between two polymorphisms (rs3800779 and rs1036145) that have been associated with the expression of a truncated and brain-specific isoform of *KCNH2*, i.e., the ratio of *KCNH2-3.1*:*KCNH2-1A* (Huffaker et al., 2009). A higher degree of *KCNH2-3.1* expression has been observed in patients with schizophrenia (Huffaker et al., 2009) and the rs3800779 SNP has been associated with schizophrenia, as well as with low IQ and working memory performance (Huffaker et al., 2009). A study of 191 Japanese individuals (Hashimoto et al., 2013) likewise showed an association between rs3800779 and working memory, as well as attention, but not with either verbal or visual memory (as assessed by the Rey Auditory Verbal Learning test and the Wechsler Memory Scale-Revised), nor with social cognition as measured by the Facial Emotion Labeling Test (Hashimoto et al., 2013).

As mentioned in the introduction, face recognition memory involves a face-specific mechanism (Rezlescu et al., 2014; Weigelt et al., 2014). The potential influence of variation in *NOS3* or *KCNH2* on social memory appears, however, not to be specific to this dimension, but also to include word pair recognition memory, as suggested by the weaker, but significant association in the exploratory study. Recent studies show overlaps in development and function of face and word recognition (Bukowski et al., 2013; Dundas et al., 2013), such that those with difficulties in recognizing faces also show deficits for words and vice versa (Behrmann and Plaut, 2014). Face and word recognition memory did however not correlate in the exploratory sample, and the genetic effects on face recognition were independent of word recognition memory, as the regression coefficient did not change when the latter was included in the model.

Except for visual category, differences in the strength of the association for words and faces in the exploratory study could be due to other dissimilarities between these two tasks. Firstly, the words were not new to the participants in the same way as the faces were, because words are already represented in long-term memory from previous exposures. This is a point that other studies have solved by using famous faces (Nie et al., 2014). Second, the word pair recognition, but not the face recognition, test involved the component process of matching the two parts of the pair, the category and the instance.

In the exploratory study, the association between the *NOS3* and *KCNH2* polymorphisms and face recognition held in both men and women, whereas the larger validation study only revealed an effect in men. Differences in the characteristics of the social memory tasks used in the two studies may possibly explain this discrepancy. The exploratory study included the recognition of the identity of faces of neutral face expressions, whereas the validation study included faces of several different emotional expressions. However, a previous study of face recognition in children showed that emotional expressions did not influence the recognition of face identity (Krebs et al., 2011). A possible mechanism explaining the sex differences is suggested by the finding that estrogen induces NO production via NOS activation in endothelial cells (Nevzati et al., 2015), and that *NOS3* polymorphisms hence may influence the degree of estrogen-induced production differently in men and women. Although there was no significant difference in face recognition performance between men and women in either of the present samples, sex differences have been repeatedly reported for both face recognition and its underlying neural mechanisms (Fischer et al., 2007).

In conclusion, by using one exploratory sample to isolate a number of promising polymorphisms and then examining them in a targeted manner in a validation sample, we have demonstrated an association between polymorphisms in the *KCNH2* and *NOS3* genes and social memory.

## Author contributions

Study concept and design: SH, DH, LW. Acquisition, analysis, or interpretation of data: All authors. Drafting of the manuscript: SH. Critical revision of the manuscript for important intellectual content: All authors. Final approval of the version to be published: All authors. Agreement to be accountable for all aspects of the work in ensuring that questions related to the accuracy or integrity of any part of the work are appropriately investigated and resolved: All authors.

### Conflict of interest statement

The authors declare that the research was conducted in the absence of any commercial or financial relationships that could be construed as a potential conflict of interest.
